# Ligand-Based Virtual Screening for Discovery of Indole Derivatives as Potent DNA Gyrase ATPase Inhibitors Active against *Mycobacterium tuberculosis* and Hit Validation by Biological Assays

**DOI:** 10.1021/acs.jcim.4c00511

**Published:** 2024-07-12

**Authors:** Bongkochawan Pakamwong, Paptawan Thongdee, Bundit Kamsri, Naruedon Phusi, Somjintana Taveepanich, Kampanart Chayajarus, Pharit Kamsri, Auradee Punkvang, Supa Hannongbua, Jidapa Sangswan, Khomson Suttisintong, Sanya Sureram, Prasat Kittakoop, Poonpilas Hongmanee, Pitak Santanirand, Jiraporn Leanpolchareanchai, James Spencer, Adrian J. Mulholland, Pornpan Pungpo

**Affiliations:** † Department of Chemistry and Center of Excellence for Innovation in Chemistry, Faculty of Science, Ubon Ratchathani University, Ubon Ratchathani 34190, Thailand; ‡ Division of Chemistry, Faculty of Science, Nakhon Phanom University, Nakhon Phanom 48000, Thailand; § Department of Chemistry, Faculty of Science, Kasetsart University, Bangkok 10900, Thailand; ∥ Department of Biological Science, Faculty of Science, Ubon Ratchathani University, Ubon Ratchathani 34190, Thailand; ⊥ National Nanotechnology Center, NSTDA, 111 Thailand Science Park, Klong Luang, Pathum Thani 12120, Thailand; # Chulabhorn Research Institute, Laksi, Bangkok 10210, Thailand; ∇ Program in Chemical Sciences, Chulabhorn Graduate Institute, Bangkok 10210, Thailand; ○ Center of Excellence on Environmental Health and Toxicology (EHT), OPS, Ministry of Higher Education, Science, Research and Innovation, Bangkok 10210, Thailand; ◆ Division of Clinical Microbiology, Department of Pathology, Faculty of Medicine, Ramathibodi Hospital, Mahidol University, Bangkok 10400, Thailand; ¶ Department of Pharmacy, Faculty of Pharmacy, Mahidol University, Bangkok 10400, Thailand; & School of Cellular and Molecular Medicine, Biomedical Sciences Building, University of Bristol, Bristol BS8 1TD, U.K.; ● Centre for Computational Chemistry, School of Chemistry, University of Bristol, Bristol BS8 1TS, U.K.

## Abstract

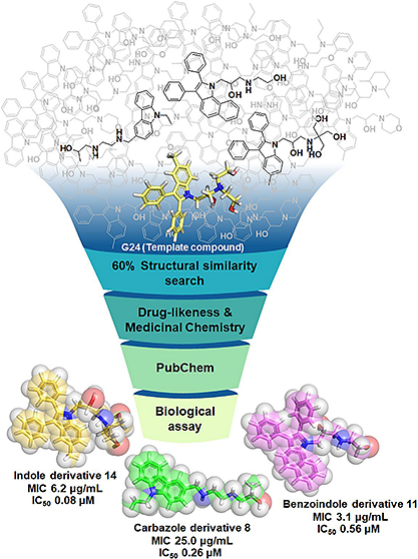

*Mycobacterium tuberculosis* is the single most important global infectious disease killer and a World Health Organization critical priority pathogen for development of new antimicrobials. *M. tuberculosis* DNA gyrase is a validated target for anti-TB agents, but those in current use target DNA breakage-reunion, rather than the ATPase activity of the GyrB subunit. Here, virtual screening, subsequently validated by whole-cell and enzyme inhibition assays, was applied to identify candidate compounds that inhibit *M. tuberculosis* GyrB ATPase activity from the Specs compound library. This approach yielded six compounds: four carbazole derivatives (**1**, **2**, **3**, and **8**), the benzoindole derivative **11**, and the indole derivative **14**. Carbazole derivatives can be considered a new scaffold for *M. tuberculosis* DNA gyrase ATPase inhibitors. IC_50_ values of compounds **8**, **11**, and **14** (0.26, 0.56, and 0.08 μM, respectively) for inhibition of *M. tuberculosis* DNA gyrase ATPase activity are 5-fold, 2-fold, and 16-fold better than the known DNA gyrase ATPase inhibitor novobiocin. MIC values of these compounds against growth of *M. tuberculosis* H37Ra are 25.0, 3.1, and 6.2 μg/mL, respectively, superior to novobiocin (MIC > 100.0 μg/mL). Molecular dynamics simulations of models of docked GyrB:inhibitor complexes suggest that hydrogen bond interactions with GyrB Asp79 are crucial for high-affinity binding of compounds **8**, **11**, and **14** to *M. tuberculosis* GyrB for inhibition of ATPase activity. These data demonstrate that virtual screening can identify known and new scaffolds that inhibit both *M. tuberculosis* DNA gyrase ATPase activity in vitro and growth of *M. tuberculosis* bacteria.

## 1 Introduction

Tuberculosis (TB), caused by the bacillus *Mycobacterium tuberculosis*, is the leading global cause of death from a single infectious agent, ranking above HIV/AIDS. In 2022, the number of people dying from TB increased to a total of 1.3 million, and an estimated 10.6 million people worldwide fell ill with TB. The global impact of TB disease, together with the increasing incidence of multidrug-resistant (MDR) strains, has led the World Health Organization to place *M. tuberculosis* in its highest (critical) category of bacterial pathogens against which new antibacterials must be developed.^1^

*Mycobacterium tuberculosis* DNA gyrase, comprising DNA gyrase A (GyrA) and DNA gyrase B (GyrB), regulates DNA topology by introducing negative supercoils into DNA using ATP as a cofactor.^2,3^ GyrA catalyzes the breakage and religation of bound DNA (the gate or G-segment), whereas GyrB both captures a second DNA segment (the translocated or T-segment) and is responsible for the hydrolysis of ATP.^4^ Based on these catalytic functions of DNA Gyrase, two binding sites, the DNA cleavage-ligation active site and the ATP binding site, have been validated as target binding sites for anti-tuberculosis agents. Fluoroquinolone (FQ) antibacterials, such as ciprofloxacin, ofloxacin, levofloxacin, and moxifloxacin, are DNA gyrase inhibitors binding to the cleavage-ligation active site and are extensively used antibiotics, serving as second-line agents in the treatment of infections caused by multidrug-resistant (MDR) tuberculosis strains.^5−10^ However, mutations close to the DNA binding site of DNA gyrase reduce the potency of fluoroquinolones.^11−16^

The development of fluoroquinolone resistance has prompted the discovery of novel compounds that interfere with an alternative catalytic function of DNA gyrase, namely the ATPase activity catalyzed by the GyrB subunit. Inhibition of ATPase activity by these compounds involves noncovalent binding to the ATP binding site. Derivatives of quinoline,^17−19^ aminopiperidine,^20^ thiazole,^21−23^ pyrrolamide,^24^ benzofurans,^25,26^ benzo[*d*]isothiazole,^25^ benzimidazole,^27^ phenylthiophene,^28,29^ and pyrimido[4,5-*b*]indol-8-amine^30,31^ scaffolds have all been identified as inhibitors of the ATPase activity of *M. tuberculosis* DNA gyrase. Furthermore, in our previous work benzoindole and indole derivatives^32,33^ have been identified as novel *M. tuberculosis* DNA gyrase ATPase inhibitors using virtual screening, and subsequently validated in biological assays. However, most of these compounds exhibited limited efficacy against the growth of *M. tuberculosis*, with minimal inhibitory concentration (MIC) values exceeding 100 μg/mL. The exceptions were the two indole derivatives **G24** and **G26**, which both showed MIC values of 12.5 μg/mL, i.e., significantly more active against *M. tuberculosis* than the known DNA gyrase inhibitor novobiocin^34^ (MIC > 100 μg/mL) which is to date the only DNA gyrase ATPase inhibitor to have reached the clinic.^35^ The cytotoxicity of indole derivative **G24** is lower than that of indole derivative **G26**, as evidenced by a maximum non-cytotoxic concentration of **G24** (25 μg/mL) 15-fold higher than for **G26** (1.63 μg/mL).

Based on these data, along with encouraging cytotoxicity results (see below) the indole derivative **G24** was considered suitable for optimization to develop more potent DNA gyrase inhibitors. However, optimization of a lead compound, involving selection of structural modifications followed by synthesis and biological assay, can be both time-consuming and budget-intensive. In contrast, virtual screening can be a cost-effective and time-saving technique for discovery of new compounds.^36−38^ This approach has been successfully used to identify multiple candidate anti-tuberculosis agents including inhibitors of PknB, PknG, DprE1, MurB, RpfB, DXPS, PimA, and CtpF.^39−46^ Based on these promising previous findings, the indole derivative **G24** was utilized as the starting template for structural similarity searches, constituting the initial step in the virtual screening approach used in the present work. These identified multiple candidate ATPase inhibitors from the compound library of the Specs database (www.specs.net). Biological evaluation, including determination of minimum inhibitory concentration (MIC) for *M. tuberculosis* growth inhibition, and half-maximal inhibitory concentration (IC_50_) for GyrB ATPase activity in vitro, showed that five of these compounds are significantly more effective for inhibition of *M. tuberculosis* growth and DNA gyrase ATPase activity than the initial indole derivative **G24**. These findings demonstrate the ability of virtual screening to identify novel inhibitors of *M. tuberculosis* DNA gyrase ATPase activity that inhibit growth of *M. tuberculosis*.

## 2 Materials and Methods

### 2.1 Virtual Screening Approach

**Figure 1:**
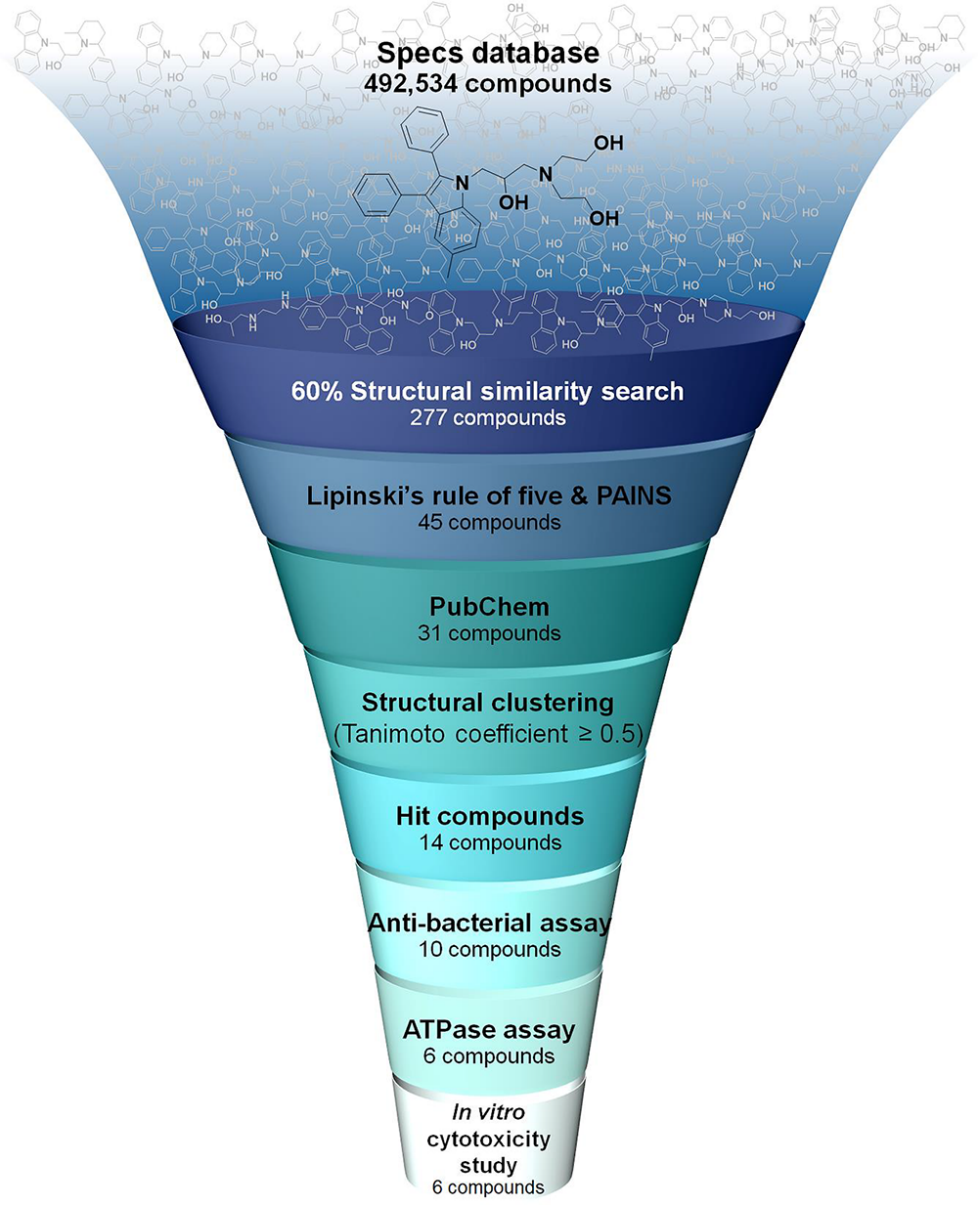
Virtual screening workflow for discovery of *M. tuberculosis* DNA gyrase inhibitors.

**Figure 2:**
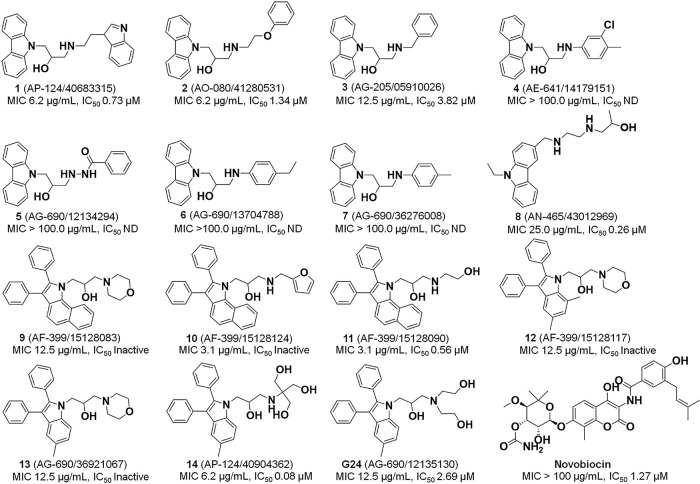
New hit compounds identified in this work. Structures of hit compounds (**1**–**14**), **G24**, and novobiocin together with Specs code, MIC against *M. tuberculosis* H37Ra, and IC_50_ for inhibition of *M. tuberculosis* DNA gyrase ATPase activity. ND indicates not determined.

Previously, our efforts aimed to identify inhibitors of the *M. tuberculosis* DNA gyrase ATPase, employing virtual screening techniques. However, due to the absence of available X-ray crystal structures of *M. tuberculosis* GyrB complexed with inhibitors, ligand-based virtual screening was limited. In one of our publications, we detailed the use of ligand-based virtual screening with bioisosteric designed compounds as templates, followed by structure-based virtual screening and biological assays. This approach resulted in a substantial percentage (78%) of hits yielding leads capable of inhibiting ATPase activity (Table S1), but none exhibited a minimal inhibitory concentration (MIC) against the growth of *M. tuberculosis* H37Ra.^32^ This outcome may be attributed to the fact that the designed template used in this study was not evaluated for inhibiting the growth of *M. tuberculosis*. Consequently, in another publication, we exclusively employed structure-based virtual screening, which marginally increased the percentage (7%) of hits to leads active against both the growth of *M. tuberculosis* and ATPase activity (Table S1).^33^ One of these identified leads, indole derivative **G24**, served as the template for ligand-based virtual screening in this study. Utilizing this compound as the template, all small molecules (492,534 compounds) in the Specs database (http://www.specs.net) were initially filtered by structural similarity search carried out on http://www.specs.net with a threshold of 60 percent (Figure 1). Filtered compounds that complied with Lipinski’s rule of five, and were not excluded by subsequent screening to exclude pan-assay interference compounds (PAINS) evaluated on http://www.swissadme.ch/index.php,^47^ were chosen for further investigation. Biological tests of these compounds against the growth of *M. tuberculosis* were subsequently checked on the PubChem web interface, an open chemistry database providing chemical information from authoritative sources (https://pubchem.ncbi.nlm.nih.gov/).^48^ Compounds displaying both active and inactive responses against *M. tuberculosis* growth in PubChem were excluded, as their biological activities have already been reported in other work. Compounds present in PubChem without recorded biological evaluation against *M. tuberculosis* growth were subjected to structural clustering using ChemMine tools.^49^ Those resembling the inactive compounds within PubChem were removed, whereas the remaining compounds were considered as potential hits (Figure 2).

### 2.2 Antimycobacterial Assay

The determination of the minimum inhibitory concentration (MIC) for the hit compounds against the growth of *M. tuberculosis* H37Ra was conducted through a microplate Alamar blue assay (MABA), as described in our previous work.^33^ Hit compounds were purchased from the vendor (www.specs.net), dissolved in DMSO (Sigma-Aldrich) and then subjected to serial two-fold dilution, resulting in final concentrations ranging from 0.1 to 100 μg/mL, maintaining a constant DMSO concentration of 0.156% v/v. A mycobacterial suspension was prepared in a solution of 0.04% Tween 80, which was subsequently diluted with sterile distilled water to attain turbidity equivalent to a McFarland standard of no. 1. Following this, the suspension was further diluted at a 1:50 ratio with Middlebrook 7H9 media containing 0.2% v/v glycerol and 1.0 g/L casitone (7H9GC). Subsequently, 100 μL of this diluted suspension was added to each well of the microplate used for the assay. After an incubation period of approximately 7 days at 37 °C, a mixture of 12.5 μL of 20% Tween 80 and 20 μL of Alamar blue (SeroTec Ltd., Oxford, UK) was added to all wells. The growth of the microorganisms was assessed after an additional pre-incubation period of 16–24 h at 37 °C, during which time a visible color shift from blue to pink indicated positive growth. The MIC is defined as the lowest concentration at which the color change was prevented. Novobiocin (Sigma-Aldrich) was used as a positive control.

### 2.3 Overexpression and Purification of *M. tuberculosis* GyrA and GyrB

The overexpression, expression, and purification of *M. tuberculosis* DNA GyrA and GyrB were detailed in our previous work.^33^ In brief, PCR-amplified fragments corresponding to the *M. tuberculosis* GyrA and GyrB subunits were individually cloned into the NdeI/XhoI restriction sites of the pET21a(+) and pET28a(+) vectors (Novagen), resulting in plasmids encoding GyrA with a C-terminal hexa-histidine tag and GyrB with an N-terminal hexa-histidine tag. Subsequently, these plasmids were transformed into *E. coli* BL21(DE3)pLysS^50^ (Novagen) using the heat-shock method to express recombinant proteins. Cultures of *E. coli* BL21(DE3) pLysS were grown in LB medium, supplemented with ampicillin for GyrA and kanamycin for GyrB. Subsequently, 0.2 mM isopropyl-β-D-thiogalactopyranoside (IPTG) was used to induce expression. Protein purification was performed using Ni-NTA beads manually packed into a 2.5 cm diameter polypropylene gravity-flow column (Expedeon). The purity of these proteins was analyzed by 10% Sodium Dodecyl Sulfate polyacrylamide gel electrophoresis (SDS-PAGE).^51^

### 2.4 Inhibition (IC_50_) Assays of *M. tuberculosis* DNA Gyrase ATPase Activity

Inhibition of *M. tuberculosis* DNA gyrase holoenzyme was measured by monitoring ATPase activity using the coupled pyruvate kinase/lactate dehydrogenase assay, consistent with our aim of identifying inhibitors of GyrB ATPase activity as opposed to agents acting upon gyrase by other mechanisms. Half-maximal inhibitory concentration (IC_50_) values for inhibition of *M. tuberculosis* DNA gyrase ATPase activity were evaluated for hit compounds as described in our previous work.^33^ Novobiocin (Sigma-Aldrich) was used as a positive control. IC_50_ determinations were carried out using data collected in triplicate, involving at least 11 inhibitor concentrations obtained from a serial 2-fold dilution, and values calculated from logIC_50_ obtained by fitting the dependence of % inhibition (obtained by normalizing data) on the log of inhibitor concentration using nonlinear regression in GraphPad Prism 8 (GraphPad Inc.).

### 2.5 In Vitro Cytotoxicity Study

Caco-2 cells (ATCC no. HTB-37) were used for cytotoxicity testing of selected compounds as described in our previous work.^32^ Cell viability of tested compounds on Caco-2 cells was determined using 3-(4,5-dimethylthiazol-2-yl)-2,5-diphenyltetrazolium bromide (MTT) following a modified assay procedure.^52^ Absorbance was measured at 590 nm using a microplate reader (Infinite M200 PRO, Tecan Group Ltd., Switzerland). The experiment was performed in triplicate and compound concentrations at which cells retained a viability of more than 80% were considered as nontoxic to cells.^53^

### 2.6 Molecular Docking Calculations

Molecular docking calculations were used to generate the initial complexes between the GyrB subunit of *M. tuberculosis* and inhibitors. These complexes were then employed in molecular dynamics (MD) simulations. The docking calculations were conducted using the Glide program^54−56^ in extra precision (XP) mode and were executed on a DELL Intel Core i5-7500 computer, as detailed in our previous work.^33^ Four X-ray crystal structures of the ATPase domain of the *M. tuberculosis* GyrB subunit are available: one in the apo form (PDB code 6GAV)^57^ and three in the holo form complexed with AMPPCP (PDB code 3ZM7)^58^ and AMPPNP (PDB codes 3ZKD and 6GAU),^57,58^ functioning as nonhydrolyzable ATP analogues. However, X-ray crystal structures of the *M. tuberculosis* GyrB subunit complexed with other inhibitors are not currently available. In contrast, structures of GyrB fragments complexed with inhibitors (PDB codes 4BAE, 6Y8O, and 4B6C)^24,31,59^ are available for *M. smegmatis*, another species of mycobacterium that does not cause tuberculosis. Due to the current unavailability of any structure of the *M. tuberculosis* GyrB subunit bound to an inhibitor, other than nonhydrolyzable ATP analogues, we utilized the GyrB 47 kDa ATPase domain structure, complexed with inhibitor **G24** and obtained from our previously described MD simulations,^33^ for the docking calculations in this study. The Protein Preparation Wizard Workflow and LigPrep module integrated within the Maestro program^60,61^ were employed for receptor and small molecule preparation, respectively. The initial 2D coordinates of all small molecules downloaded from the Specs database were generated as 3D structures (mol2 format) using the LigPrep module and subsequently used for molecular docking calculations. The docking grid box was assigned using the default protocol and centered upon inhibitor **G24**.

### 2.7 Molecular Dynamic Simulations

The initial inhibitor/enzyme complex structures generated from molecular docking calculations were used for MD simulations using AMBER20 software^62^ on high performance computing GPU hardware as described in our previous work.^33^ The Amber ff14SB force field was used for GyrB, and the general Amber force field (GAFF)^63^ and restrained electrostatic potential (RESP) partial charges^64−66^ were used for inhibitors. The initial complex structure was solvated with TIP3P water molecules^67^ in a cubic box extending 10 Å from the solute species. Na^+^ ions were added to neutralize the system. Energy minimization was performed using a steepest descent algorithm followed by a conjugate gradient algorithm. This was followed by 70 ps of position-restrained dynamics simulation with a restraining weight of 2 kcal/mol Å^2^ at 300 K under an isobaric condition. Finally, three replicate 200 ns MD simulations were performed with no restraints using the same conditions. The cpptraj module^68^ in AMBER20 was employed for the root-mean-square deviation (RMSD) calculations, for analysis of hydrogen bonding and clustering of the snapshots collected from the equilibrium state of each system.

### 2.8 Binding Free Energy Calculation

Molecular mechanics Poisson–Boltzmann surface area (MM/PBSA) and molecular mechanics Generalized Born surface area (MM/GBSA) methods^69^ as described in our previous work^33^ were used to estimate the binding free energies (Δ*G*_PBSA_ and Δ*G*_GBSA_, respectively) of the receptor–ligand complexes obtained from MD simulations. Snapshots extracted every 40 ps over the equilibrium state of the MD simulation of each receptor–ligand complex were used to calculate Δ*G*_PBSA_ and Δ*G*_GBSA_. The entropy contribution was excluded from the calculations of Δ*G*_PBSA_ and Δ*G*_GBSA_, due to possibility of introduction of additional errors.^70,71^

### 2.9 Pairwise Energy Decomposition

Pairwise energy decomposition was employed to calculate the interaction energies between pairs of *M. tuberculosis* GyrB residues and inhibitors in the enzyme–inhibitor complexes. This approach was utilized to quantitatively analyze the contribution of individual residues in the *M. tuberculosis* GyrB binding pocket to inhibitor binding. The python script (MMPBSA.py)^72^ in the AMBER20 program was used to perform pairwise energy decomposition (idecomp = 4) using the Molecular Mechanics-Generalized Born Surface Area (MM-GBSA) method. Snapshots extracted every 40 ps over the equilibrium state of the MD simulation of enzyme–inhibitor complex were used for energy decomposition. The pairwise decomposition energy includes the gas-phase and solvation interaction energies but does not include the entropic contribution. The gas-phase interaction energies, including the van der Waals (Δ*G*_vdW_) and electrostatic (Δ*G*_ele_) contributions, were computed using the Sander program in AMBER20. The solvation interaction energies, including the contributions of polar (Δ*G*_ele,sol_) and non-polar solvation (Δ*G*_nonpol,sol_), were calculated by using the generalized Born (GB) model (GB^OBC^ model II, igb = 5)^73^ and the solvent accessible surface area (SASA) method,^74^ respectively.

## 3 Results and Discussion

### 3.1 Ligand-Based Virtual Screening

The ligand-based virtual screening workflow employed here for discovery of *M. tuberculosis* DNA gyrase ATPase inhibitors is shown in Figure 1. Initially, a structural similarity search (at 60% similarity) using compound **G24** as the template was used to select 277 compounds from the Specs database (total 492,534 compounds). The results were then filtered by application of Lipinski’s rule of five^47,75^ and PAINS^76^ filtering, to yield 40-five compounds. The PubChem web interface was then interrogated to establish the extent of prior biological activity data for these compounds. At the time of enquiry PubChem reported activity assay results against *M. tuberculosis* growth for 14 compounds, of which three were active and 11 inactive (Table S2), while results for 30-one compounds were not reported. Thus, all 14 previously tested compounds were removed from further investigation, while the remaining 30-one compounds were used for structural clustering. Seventeen of these 30-one compounds that resembled the 11 inactive compounds (Tanimoto coefficient ≥0.5) were removed, yielding 14 hit compounds (Figure 2). These hit compounds are classified into three groups including carbazole derivatives (compounds **1**–**8**), benzoindole derivatives (compounds **9**–**11**), and indole derivatives (compounds **12**–**14**). Previously described inhibitors of *M. tuberculosis* DNA gyrase ATPase activity include quinoline,^17−19^ aminopiperidine,^20^ thiazole,^21−23^ pyrrolamide,^24^ benzofurans,^25,26^ benzo[*d*]isothiazole,^25^ benzimidazole,^27^ phenylthiophene,^28,29^ and pyrimido[4,5-*b*]indol-8-amine^30,31^ scaffolds, as well as benzoindole^32^ and indole derivatives (**G24** and **G26**)^33^ as we recently identified. Thus, carbazole derivatives (compounds **1**–**8**) can be considered a new (previously unreported) class of *M. tuberculosis* DNA gyrase ATPase inhibitors.

### 3.2 Biological Assays

MIC values for the 14 hit compounds were evaluated against the avirulent H37Ra *M. tuberculosis* strain. Ten of the 14 hits showed MIC values in the range 3.1–25.0 μg/mL. These results indicate that these ten compounds are significantly more active against *M. tuberculosis* than the well-characterized DNA gyrase ATPase inhibitor novobiocin^34^ (MIC > 100 μg/mL; Figure 2). While novobiocin is considered a weaker ATPase inhibitor than the alternative natural aminocoumarins clorobiocin and coumermycin^77,78^; the MIC values above compare favorably with those reported for *M. tuberculosis* growth inhibition by coumermycin A1 (2.5 μg/mL^79^), particularly when considering the distinct mode of action of coumermycin A1, that binds simultaneously to two GyrB ATPase sites.^80^

**Figure 3:**
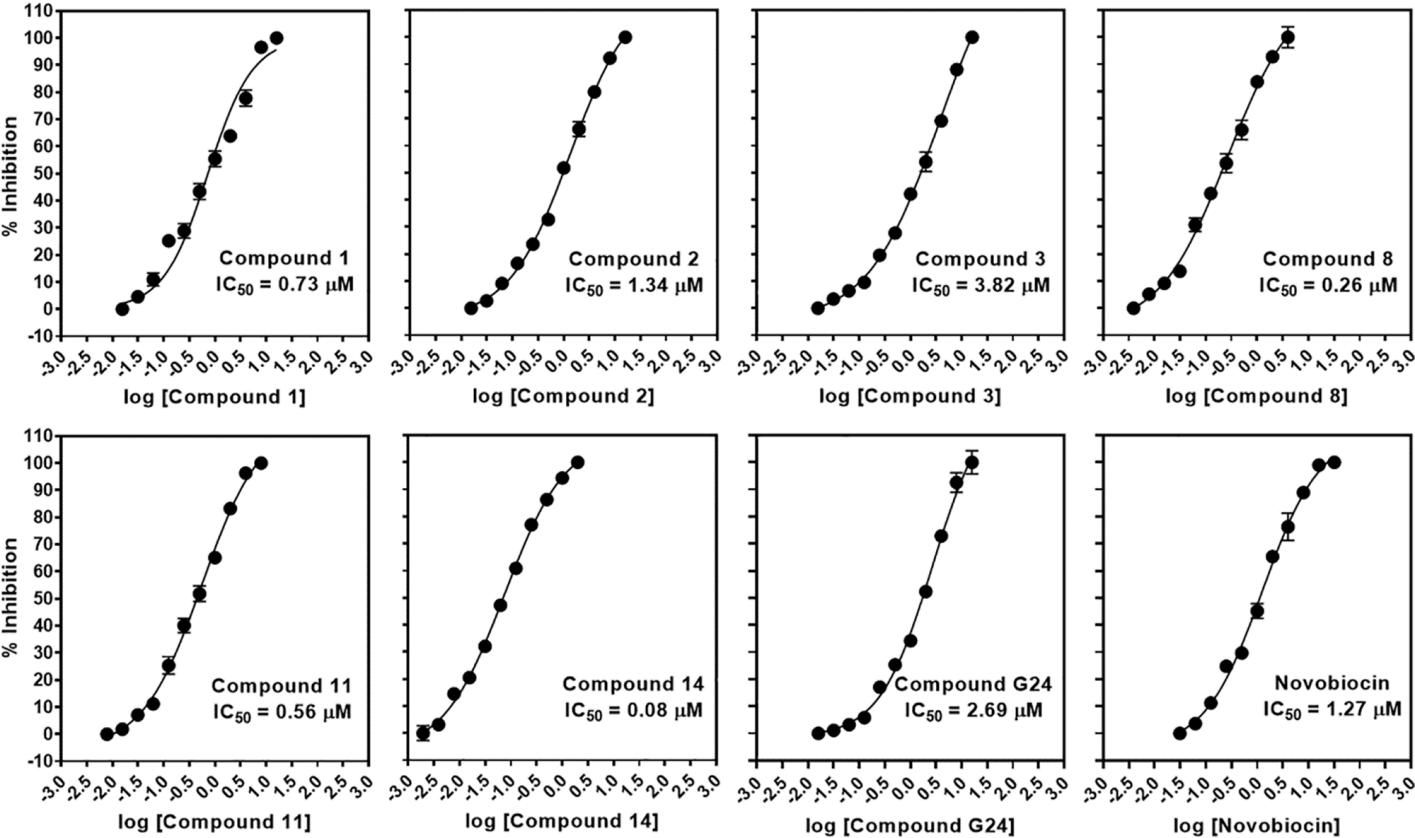
Inhibition of *M. tuberculosis* DNA gyrase ATPase activity. IC_50_ curves for inhibition of *M. tuberculosis* DNA gyrase ATPase activity by compounds **1**, **2**, **3**, **8**, **11**, **14**, **G24**, and novobiocin. IC_50_ values were obtained from nonlinear regression fitting of % inhibition to log of the inhibitor concentration. Error bars represent standard deviations (*N* = 3). Note that in some cases, these are obscured by symbols. Further details of fit parameters are given in Table S3.

Next, IC_50_ values for all ten active compounds were evaluated for inhibition of *M. tuberculosis* DNA gyrase ATPase activity, to verify this as their target enzyme. Six of the ten tested compounds (**1**, **2**, **3**, **8**, **11**, and **14**) inhibited *M. tuberculosis* DNA gyrase ATPase activity, with IC_50_ values 0.73, 1.34, 3.82, 0.26, 0.56, and 0.08 μM, respectively (Figure 3; details of fits are provided in Table S3). In comparison, the IC_50_ value for novobiocin was measured to be 1.27 μM (Figure 3), within the range of values for ATPase inhibition reported by others (0.02^30^; 0.21^31^; 4.21 μM^81^) using various assay methodologies and *M. tuberculosis* gyrase constructs. These data demonstrate that six of the identified compounds are active in both whole cell growth and enzyme inhibition assays and suggest that their anti-tubercular activity is likely to arise from inhibition of the ATPase activity of *M. tuberculosis* DNA gyrase.

### 3.3 Cytotoxicity of Lead Compounds

The six lead compounds, including four carbazole derivatives (**1**, **2**, **3**, and **8**), the benzoindole derivative **11**, and the indole derivative **14,** that showed activities against *M. tuberculosis* growth and DNA gyrase ATPase activity, were selected for evaluation of their cytotoxicity toward Caco-2 cells. All of the compounds were non-cytotoxic toward Caco-2 cells at maximum concentrations of 25.0, 12.5, 12.5, 12.5, 0.87, and 12.5 μg/mL, respectively (Figure S1). Two carbazole derivatives (**1** and **2**) and the indole derivative **14** display maximum non-cytotoxic concentrations (25.0, 12.5, and 12.5 μg/mL, respectively) higher than their MIC values (6.2, 6.2, and 3.1 μg/mL, respectively). The maximum non-cytotoxic concentration of the carbazole derivative **3** is equivalent to the MIC value of 12.5 μg/mL, whereas those of carbazole derivative **8** and benzoindole derivative **11** (12.5 and 0.87 μg/mL, respectively) are lower than the MIC values (25.0 and 3.1 μg/mL, respectively).

### 3.4 Interactions of Inhibitors with *M. tuberculosis* DNA Gyrase B

**Figure 4:**
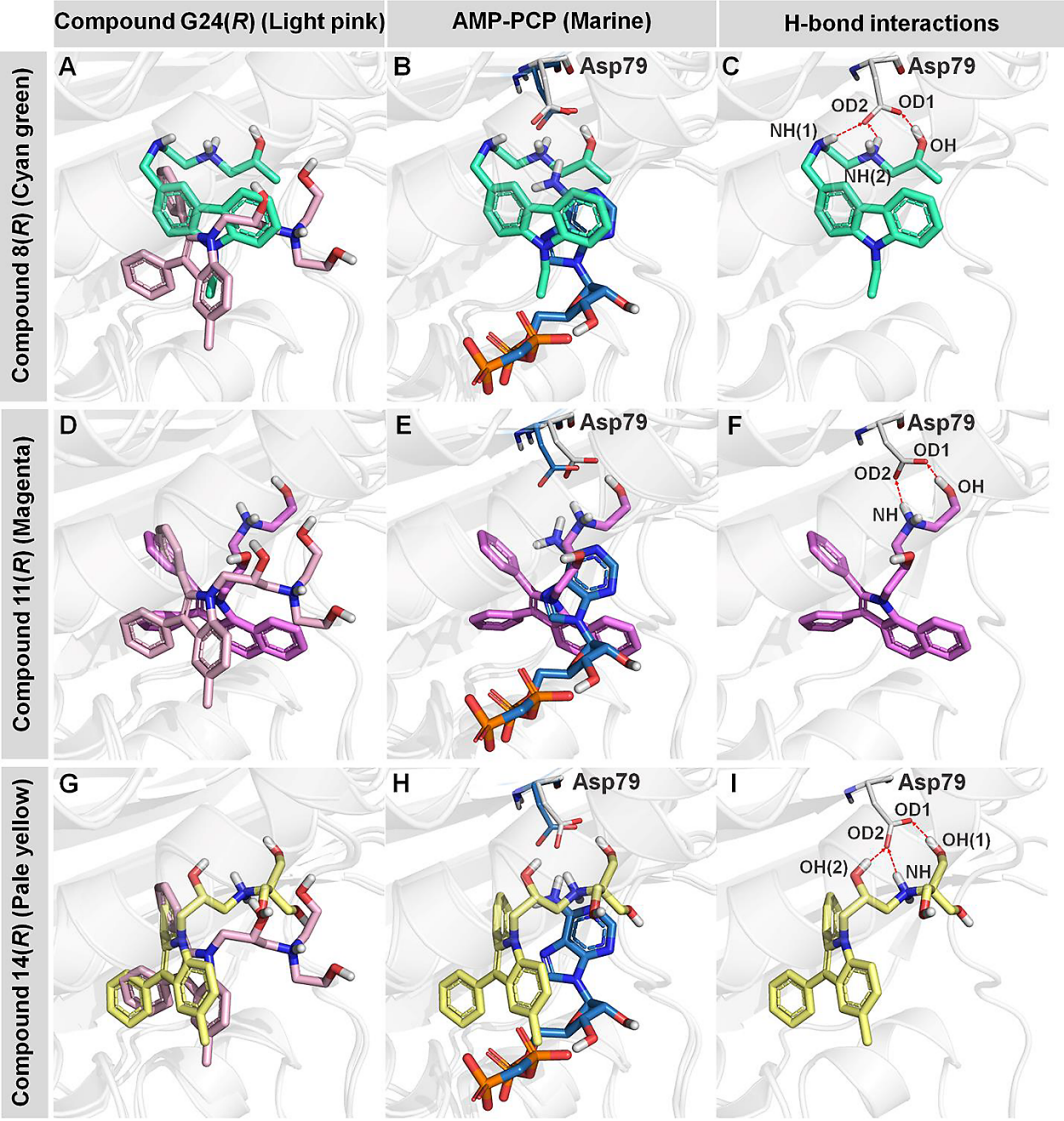
Superimpositions of AMP-PCP, indole derivatives **G24,** and compounds **8(*R*)**, **11(*R*)**, and **14(*R*)** in the *M. tuberculosis* DNA gyrase ATP binding site. (A), (D), and (G) Superimpositions of previously described indole derivative (**G24**, R configuration, light pink stick) and compounds **8(*****R*****)**, **11(*****R*****)**, and **14(*****R*****)** (cyan green, magenta, and pale yellow sticks) in the *M. tuberculosis* GyrB ATP binding site. Binding modes are predicted from MD simulations. (B), (E), and (H) Superimposition of AMP-PCP (PDB 3ZM7,^58^ marine sticks) and compounds **8(*R*)**, **11(*R*)**, and **14(*R*)** (cyan green, magenta, and pale yellow sticks, binding modes predicted from MD simulations). (C), (F), and (I) Hydrogen bond interactions (dashed red arrows) of compounds **8(*R*)**, **11(*R*)**, and **14(*R*)** with Asp79.

Compound **8**, the carbazole derivative showing the most potent inhibition of *M. tuberculosis* DNA gyrase ATPase activity, was selected for analysis of its interactions with the *M. tuberculosis* GyrB ATP binding site using molecular dynamics (MD) simulations. The benzoindole derivative **11** and the indole derivative **14**, that were also active against *M. tuberculosis* DNA gyrase ATPase activity, were also selected for further analysis using MD simulations. As all of these are chiral compounds, the binding modes of compounds **8** (carbazole derivative), **11** (benzoindole derivative), and **14** (indole derivative) were considered in both the *R* and *S* configurations. The stabilities of MD simulations of the complexes with each compound, with initial structures generated by molecular docking using Glide XP, are provided in Supporting Information (Figure S2). The binding free energies of these compounds to the GyrB 47 kDa fragment, in both stereochemical configurations, in their equilibrium states, were then calculated using the Δ*G*_PBSA_ and Δ*G*_GBSA_ methods (Table 1). The average binding free energies calculated for the carbazole derivative **8**, the benzoindole derivative **11**, and the indole derivative **14** binding to the *M. tuberculosis* DNA gyrase GyrB ATP site in the *R* configuration are significantly lower than those obtained for the *S* configuration, indicating the *R* stereomer to be the higher affinity configuration in each case. The binding modes of compounds **8**, **11**, and **14**, each in the *R* configuration, were then analyzed and compared. The binding modes for these three compounds resemble that of our previously described indole derivative **G24**(33) (Figure 4A,D,G). Further, compounds **8**, **11**, and **14** also all partially overlap with the adenosine group of the ATP analogue AMP-PCP when complexed with *M. tuberculosis* GyrB (PDB code 3ZM7).^58^ Notably, the hydrogen bond donating moieties (hydroxyl and amine groups) of these compounds are located near the carboxylate side chain of Asp79, the key residue forming a hydrogen bond interaction with the adenine NH_2_ group of AMP-PCP (Figure 4B,E,H). Hydrogen bond analysis performed on the MD simulation trajectories of the carbazole derivative **8**, benzoindole derivative **11**, and indole derivative **14** (Table S4) showed that these compounds can all form hydrogen bonds with the carboxylate oxygen atoms of Asp79 (Figure 4C,F,I), with occupancies more than 60% (Table S4). The contribution of this residue to the binding of compounds **8**, **11**, and **14** was then further investigated using decomposition energy calculations. The results showed that Asp79 is the GyrB residue that makes the most prominent contribution to the binding of all of these compounds to *M. tuberculosis* DNA gyrase GyrB, as evidenced by the low decomposition energy values of −30.2 ± 2.0, −19.9 ± 0.6, and −29.8 ± 0.1 kcal/mol, respectively (Figure S3). Thus, hydrogen bond interactions with Asp79 appear crucial for binding of all three compounds to the *M. tuberculosis* DNA gyrase GyrB subunit. Further, MD simulations were conducted on GyrB with the Asp79Ala (D79A) mutation, bound with compounds **8**, **11**, and **14** in the *R* configuration, to support the role of Asp79. The mutation from Asp79 to Ala79 disrupts the hydrogen bonds of these compounds to Asp79 in wild-type GyrB. This disruption is evidenced by the lower contribution of Ala79 to the binding of compounds **8**, **11**, and **14** with the higher decomposition energy values of −0.1, 0.1, and 0.1 kcal/mol, respectively (Figure S3). Further, the calculated binding free energies of these compounds to the mutant GyrB subunit using the Δ*G*_GBSA_ and Δ*G*_PBSA_ methods are significantly higher than those of the wild-type GyrB subunit (Table S5). These results indicate the diminished affinity binding of compounds **8**, **11**, and **14** for the D79A mutant GyrB subunit, emphasizing the significance of Asp79 as a key residue for high-affinity binding to the *M. tuberculosis* DNA gyrase GyrB subunit. Similar hydrogen bond interactions with Asp79 are also observed in the binding of three known *M. tuberculosis* DNA gyrase ATPase inhibitors, pyrrolamide (PDB code 4BAE),^24^ aminopyrazinamide (PDB code 4B6C),^59^ and novobiocin (PDB code 6Y8O)^31^ as shown in Figure S4. Further, these interactions are observed in the binding of DNA gyrase ATPase inhibitors against other bacteria such as *S. aureus*,^82−84^*E. coli*,^85−87^ and *S. pneumoniae*.^88^ Notably, the IC_50_ values for inhibition of *M. tuberculosis* DNA gyrase ATPase activity by compounds **8**, **11**, and **14** (0.26, 0.56, and 0.08 μM, respectively) that are all predicted to form hydrogen bonds with Asp79, were superior to that obtained for the indole derivative **G24** (IC_50_ 2.69 μM, Figure 3) that in previous work was not predicted to form a hydrogen bond with Asp79.^33^ These results identify Asp79 as a key residue for high-affinity binding of all three compounds to the *M. tuberculosis* DNA gyrase GyrB subunit, and consequent inhibition of its ATPase activity. In further support of this conclusion, we note that compounds **9**, **10**, **12**, and **13**, all of which lack a hydrogen bond donor at the terminus of their aliphatic substituent groups, are all inactive in ATPase inhibition assays.

**Table 1:** Binding Free Energy (Δ*G*_GBSA_ and Δ*G*_PBSA_) Calculations from MD Simulations for Lead Compounds

compound	Δ*G*_GBSA_ (kcal/mol)				Δ*G*_PBSA_ (kcal/mol)			
	replicate			average^a^	replicate			average^a^
	1	2	3		1	2	3	
**8***^R^*	–57.5	–58.9	–61.1	–59.1 ± 1.8	–46.2	–48.5	–50.4	–48.3 ± 2.1
**8***^S^*	–48.4	–49.3	–48.6	–48.8 ± 0.5	–48.4	–48.5	–46.7	–47.9 ± 1.0
**11***^R^*	–64.8	–60.7	–61.4	–62.3 ± 2.2	–57.1	–55.3	–56.1	–56.2 ± 0.9
**11***^S^*	–55.3	–50.1	–50.5	–52.0 ± 2.9	–56.6	–52.8	–53.4	–54.3 ± 2.0
**14***^R^*	–72.0	–73.0	–71.8	–72.3 ± 0.6	–62.0	–62.8	–62.0	–62.3 ± 0.5
**14***^S^*	–62.3	–62.7	–62.6	–62.5 ± 0.2	–58.1	–58.0	–57.5	–57.8 ± 0.3

a Errors are estimated from the average and the difference between the results from three separate MD simulations for each compound. *R* and *S* represent *R* and *S* configurations.

### 3.5 Structure–Activity Relationship (SAR)

We utilized our previously discovered indole derivative **G24**(33) as the template for virtual screening, leading to the identification of the indole derivative **14** in the present work. The structural similarity between the indole derivatives **G24** and **14** is their 1-(5-methyl-2,3-diphenyl-1*H*-indol-1-yl)propan-2-ol core, whereas the two compounds are distinguished by differences in their hydroxyalkyl amine substituents (Figure 2). Due to this structural difference, compound **14** exhibited improved IC_50_ and MIC values compared to **G24**. This identifies the hydroxyalkyl amine substituent as important for enhancing the activity of indole derivative **14** against both *M. tuberculosis* growth and DNA gyrase ATPase activity. The mode of binding of indole derivative **14** obtained from MD simulations reveals that this substituent is utilized for forming hydrogen bond interactions with Asp79, the key residue for binding to DNA gyrase GyrB subunit. The significance of the hydroxyalkyl amine substituent in mediating the inhibition of DNA gyrase ATPase activity is evidenced by the IC_50_ values of the indole derivatives **12** and **13** (Figure 2). These derivatives lack the hydroxyalkyl amine substituent, and any terminal hydrogen bond donating group at the termini of their equivalent substituents. The absence of any observed IC_50_ values may then result from the consequent impaired ability of these compounds to make hydrogen bonds to Asp79. Furthermore, the equivalent substituent is also a determinant of ATPase inhibitory activity (IC_50_ values) for the benzoindole derivatives also identified in the present work. Specifically, the benzoindole derivative **11**, which presents a hydroxyalkyl amine group, yielded a measurable IC_50_ value, whereas IC_50_ values for ATPase inhibition could not be determined for the benzoindole derivatives **9** and **10**. Of interest, however, the benzoindole and indole derivatives (**9**, **10**, **12**, and **13**) that lack the hydroxyalkyl amine substituent all gave MIC values for inhibition of *M. tuberculosis* growth, despite the absence of observable ATPase inhibition, suggesting that for these compounds anti-tubercular activity is unlikely to arise from inhibition of DNA gyrase GyrB ATPase activity. In contrast, the benzoindole derivative **11** and the indole derivative **14** that present the hydroxyalkyl amine substituent are active against both *M. tuberculosis* growth and DNA gyrase ATPase activity, supporting the latter as the likely basis of their anti-tubercular activity.

The carbazole derivatives identified in the present work represent a new class of *M. tuberculosis* DNA gyrase ATPase inhibitors. The carbazole derivatives **1**–**3** and **8** are all active against both *M. tuberculosis* growth and DNA gyrase ATPase activity, whereas the carbazole derivatives **4**–**7** showed no anti-tubercular activity (MIC values >100 μg/mL) and their IC_50_ values for ATPase inhibition were consequently not determined (Figure 2). The carbazole derivatives **1**–**7** contain equivalent 1-amino-3-(9*H*-carbazol-9-yl)propan-2-ol cores. Remarkably, carbazole derivatives **1**–**3**, that present methyl, ethyl, or oxyethyl linkers connecting the aliphatic amine to the terminal aromatic ring are all active against *M. tuberculosis* growth, whereas carbazole derivatives **4**–**7** with alternative linkers are not. These results indicate that the alkyl linker in the carbazole derivatives **1**–**3** is important to activity against *M. tuberculosis* growth. The carbazole derivative **8**, that possesses a chemical structure different to those of the other carbazole derivatives tested, contains a hydroxyalkyl amine substituent (Figure 2). This compound inhibited *M. tuberculosis* DNA gyrase ATPase activity approximately 3, 5, and 15-fold more effectively than the carbazole derivatives **1**–**3**, respectively. The mode of binding of carbazole derivative **8** obtained from MD simulations reveals that this substituent can be utilized to form hydrogen bond interactions with Asp79, as also observed in the binding of both the benzoindole and indole derivatives (Figure 4). Thus, across all three compound classes (carbazole, benzoindole, and indole derivatives) studied here, the hydroxyalkyl amine substituent is important to inhibitory activity toward the ATPase activity of *M. tuberculosis* DNA gyrase.

## 4 Conclusions

Ligand-based virtual screening supported by subsequent whole cell and enzyme inhibition assays identified six compounds that showed anti-tubercular activity and inhibition of the ATPase activity of *M. tuberculosis* DNA gyrase. This approach yielded a higher percentage (43%) of hits to leads active against both the growth of *M. tuberculosis* and ATPase activity compared to our two previous works.^32,33^ The identified lead compounds are classified as carbazole, benzoindole, and indole derivatives, of which carbazole derivatives represent a new scaffold for inhibition of *M. tuberculosis* DNA gyrase ATPase activity. The complexes of carbazole, benzoindole, and indole derivatives bound to the ATP binding site of the *M. tuberculosis* DNA gyrase GyrB subunit, modeled by MD simulations, highlighted the critical role of hydrogen bond interactions with Asp79 in binding. SAR analysis suggests that the hydroxyalkyl amine substituents of the benzoindole and indole derivatives, that our models implicate in hydrogen bonding to Asp79, are important to inhibition of DNA gyrase ATPase activity. In the case of the carbazole derivatives, the equivalent hydroxyalkyl amine substituent similarly enhances IC_50_ values for inhibition of DNA gyrase ATPase activity, while an alkyl linker appears crucial to inhibition of *M. tuberculosis* growth. The importance to inhibitor binding of the conserved Asp79 is noteworthy given the involvement of this residue in interactions with ATP; previous investigations identify that substitution of the equivalent position in *E. coli* reduces susceptibility to novobiocin inhibition, but is sufficiently detrimental to ATPase activity for selection of such mutations in vivo to be considered unlikely.^89^ Thus, ATPase inhibitors that, as here, derive much of their potency from hydrogen bonding to Asp79 might be at reduced risk of failure through mutational resistance. Taken together, our findings support similarity-based virtual screening as a valid and efficient tool with which to improve inhibitors of *M. tuberculosis* DNA gyrase ATPase activity, that retains the ability to identify new scaffolds able to inhibit this validated, but clinically under-exploited, target.

## Data Availability

All chemical structures in SDF format of small molecules were downloaded from Specs database (https://www.specs.net/). PAINS and physicochemical properties of these molecules were calculated by SwissADME (http://www.swissadme.ch/index.php).^47^ 2D chemical structures of small molecules in Figure 2 were generated using ChemDraw 20.1.1 (CambridgeSoft, http://www.cambridgesoft.com). IC_50_ values were calculated using GraphPad Prism8 with free trial license (GraphPad Inc., https://www.graphpad.com/) in Figure 3. Figures 4 and S4 were generated using pyMOL2.2.0 (https://pymol.org/2/).

## Supporting Information Available

Hit compound cytotoxicity against Caco-2 cells; cells were incubated with different concentrations of compounds (**1**, **2**, **3**, **8**, **11**, **14**, **G24**, and **G26**) for 24 h and viability measured by MTT assay; all-atom RMSD plots for MD simulations of *M. tuberculosis* GyrB:inhibitor complexes; per-residue analysis of inhibitor:GyrB interactions; hydrogen bond interactions of known ATPase inhibitors with Asp79 in the *M. smegmatis* DNA gyrase ATP binding site; hits and leads obtained from three different virtual screening methods; previously characterized compounds identified from virtual similarity screening; details of fit parameters for IC_50_ determinations; hydrogen bonding of most active compounds (*R*-stereomers) to GyrB Asp79; and calculated binding free energies for binding of compounds **8**, **11**, and **14** to the wild type and mutant (D79A) GyrB subunits (PDF)

## Abbreviations

TB: tuberculosis
MDR-TB: multidrug-resistant TB
*M. tuberculosis*: *Mycobacterium tuberculosis*
FQ: fluoroquinolones
GyrA: DNA gyrase A
GyrB: DNA gyrase B
MIC: minimal inhibitory concentration
IC_50_: half-maximal inhibitory concentration
PAINS: pan-assay interference compounds
MABA: microplate alamar blue assay
DMSO: dimethyl sulfoxide
PCR: polymerase chain reaction
*E. coli*: *Escherichia coli*
LB: Luria Broth
IPTG: isopropyl-β-D-thiogalactopyranoside
Ni-NTA: nickel-nitrilotriacetic acid
SDS-PAGE: sodium dodecyl sulfate polyacrylamide gel electrophoresis
MTT: 3-(4,5-dimethylthiazol-2-yl)-2,5-diphenyltetrazolium bromide
nm: nanometer
MD: molecular dynamics simulations
XP: extra precision
GPU: graphics processing unit
GAFF: general amber force field
RESP: restrained electrostatic potential
RMSD: root-mean square deviation
MM/PBSA: molecular mechanics Poisson–Boltzmann surface area
MM/GBSA: molecular mechanics generalized born surface area
SASA: Solvent accessible surface area
*S. aureus*: *Staphylococcus aureus*
*S. pneumoniae*: *Streptococcus pneumoniae*
SAR: structure–activity relationship
