# Supplementary material for: Ligand-Based Virtual Screening for Discovery of Indole Derivatives as Potent DNA Gyrase ATPase Inhibitors Active against Mycobacterium tuberculosis and Hit Validation by Biological Assays
Source: J Chem Inf Model. 2024 Jul 12;64(15):5991–6002. doi: 10.1021/acs.jcim.4c00511 (PMC11323271; doi:10.1021/acs.jcim.4c00511)
Supplement: Supplementary file 1 — ci4c00511_si_001.pdf [file ci4c00511_si_001.pdf]

Supporting Information

Ligand–Based Virtual Screening for Discovery of Indole Derivatives as Potent DNA Gyrase ATPase Inhibitors active against *Mycobacterium tuberculosis* and Hit Validation by Biological Assays

Bongkochawan Pakamwong[1], Paptawan Thongdee[1], Bundit Kamsri[1], Naruedon Phusi[1], Somjintana Taveepanich[1], Kampanart Chayajarus[1], Pharit Kamsri[2], Auradee Punkvang[2], Supa Hannongbua[3], Jidapa Sangswan[4], Khomson Suttisintong[5], Sanya Sureram[6], Prasat Kittakoop[6,7,8], Poonpilas Hongmanee[9], Pitak Santanirand[9], Jiraporn Leanpolchareanchai[10], James Spencer[11], Adrian J. Mulholland[12], Pornpan Pungpo[1,*]

[1] *Department of Chemistry and Center of Excellence for Innovation in Chemistry, Faculty of Science, Ubon Ratchathani University, Ubon Ratchathani 34190, Thailand*

[2] *Division of Chemistry, Faculty of Science, Nakhon Phanom University, Nakhon Phanom 48000, Thailand*

[3] *Department of Chemistry, Faculty of Science, Kasetsart University, Bangkok 10900, Thailand*

[4] *Department of Biological Science, Faculty of Science, Ubon Ratchathani University, Ubon Ratchathani 34190, Thailand*

[5] *National Nanotechnology Center, NSTDA, 111 Thailand Science Park, Klong Luang, Pathum Thani 12120, Thailand*

[6] *Chulabhorn Research Institute, Laksi, Bangkok 10210, Thailand*

[7] *Chulabhorn Graduate Institute, Program in Chemical Sciences, Bangkok 10210, Thailand*

[8] *Center of Excellence on Environmental Health and Toxicology (EHT), OPS, Ministry of Higher Education, Science, Research and Innovation, Bangkok 10210, Thailand*

[9] *Division of Clinical Microbiology, Department of Pathology, Faculty of Medicine, Ramathibodi Hospital, Mahidol University, Bangkok 10400, Thailand*

[10] *Department of Pharmacy, Faculty of Pharmacy, Mahidol University, Bangkok 10400, Thailand*

[11] *School of Cellular and Molecular Medicine, Biomedical Sciences Building, University of Bristol, Bristol BS8 1TD, United Kingdom*

[12] *Centre for Computational Chemistry, School of Chemistry, University of Bristol, Bristol BS8 1TS, United Kingdom*

Corresponding author

Pornpan Pungpo,

Department of Chemistry and Center of Excellence for Innovation in Chemistry,

Faculty of Science,

Ubon Ratchathani University,

Ubonratchathani, Thailand

E-mail: pornpan_ubu@yahoo.com

Fax: +664 5288379; Tel: +664 535 3400 ext. 4124

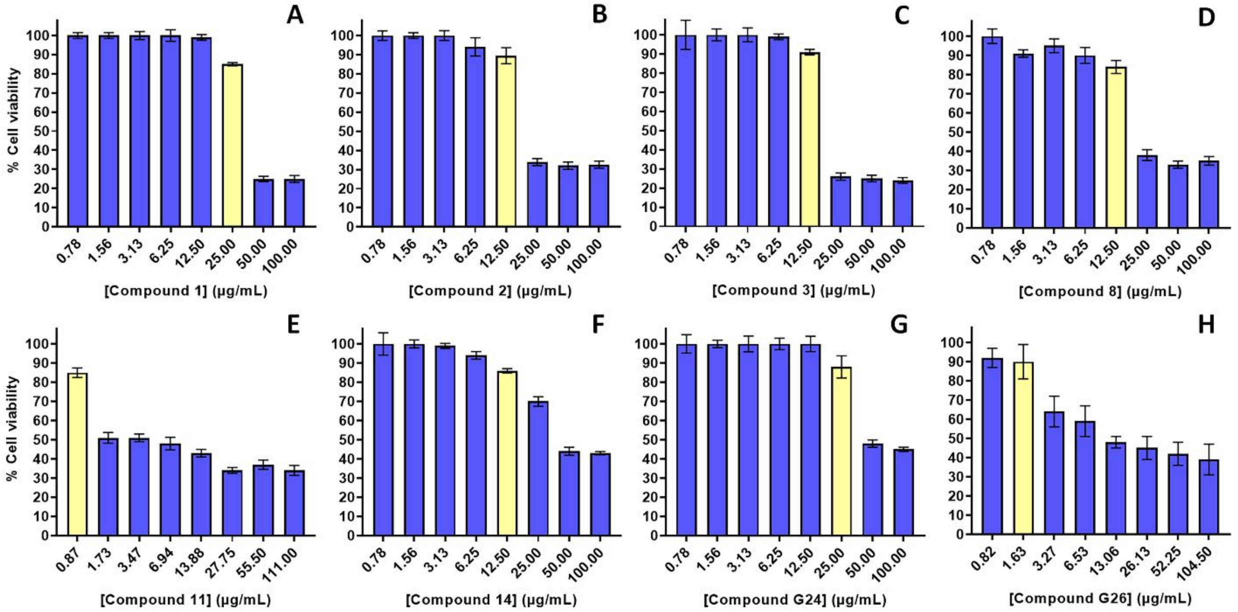

Figure S1. Hit compound cytotoxicity against Caco-2 cells. Cells were incubated with different concentrations of compounds (**1** (A), **2** (B), **3** (C), **8** (D), **11** (E), **14** (F), **G24** (G), and **G26** (H)) for 24 h and viability measured by MTT assay. Data are presented as the mean ± SD (n = 6). The yellow bars represent the maximum concentrations at which compounds are non-cytotoxic towards Caco-2 cells.

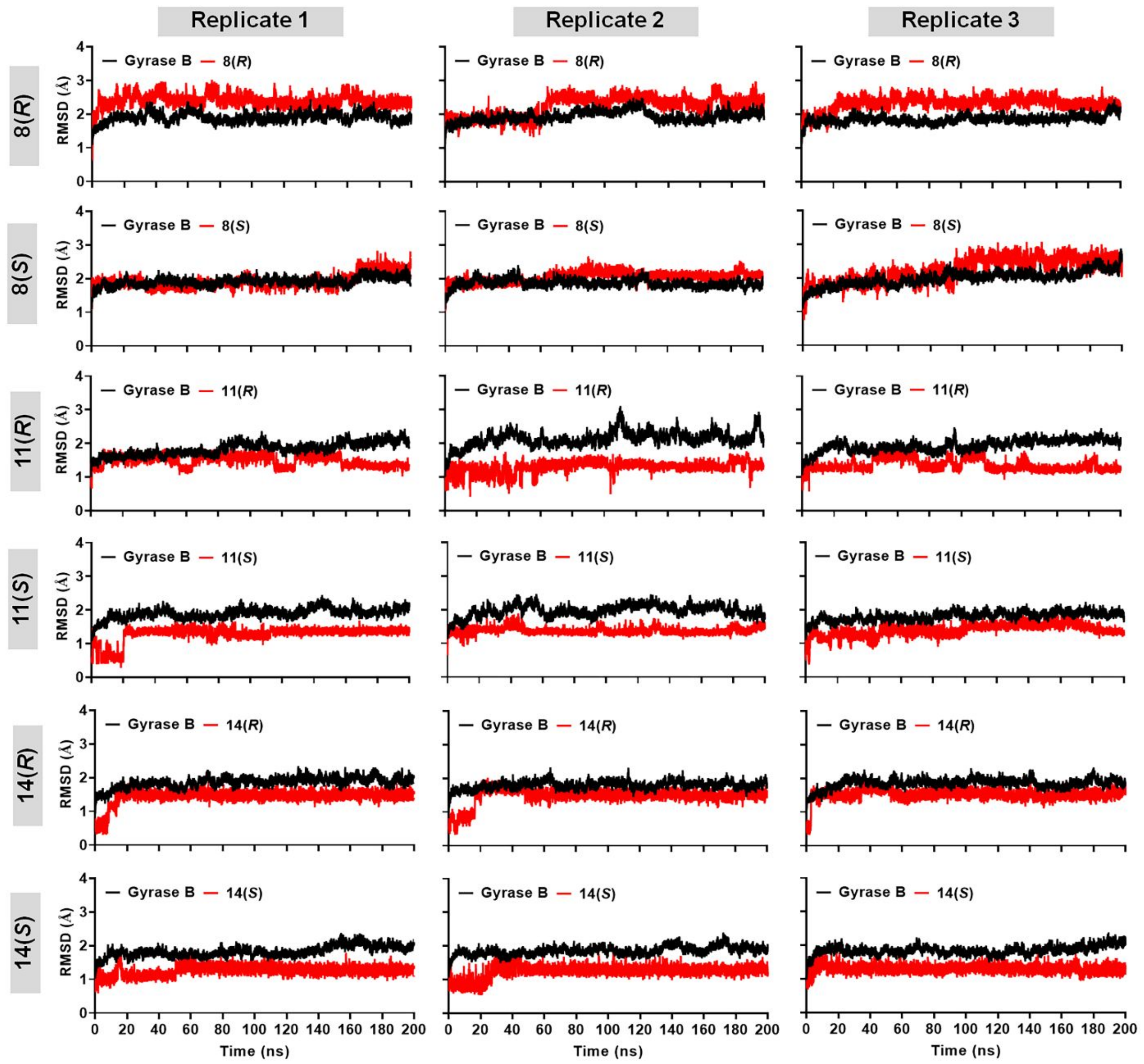

Figure S2. All-atom RMSD plots for MD simulations of *M. tuberculosis* GyrB (47 KDa fragment):inhibitor complexes. RMSD values, with respect to the initial structures obtained from molecular docking calculations, for the six systems **8**: *M. tuberculosis* DNA gyrase GyrB, **11**: *M. tuberculosis* DNA gyrase GyrB, and **14**: *M. tuberculosis* DNA gyrase GyrB (each in the *R*- and *S*- configurations), were calculated over three replicates each of 200 ns MD simulation time.

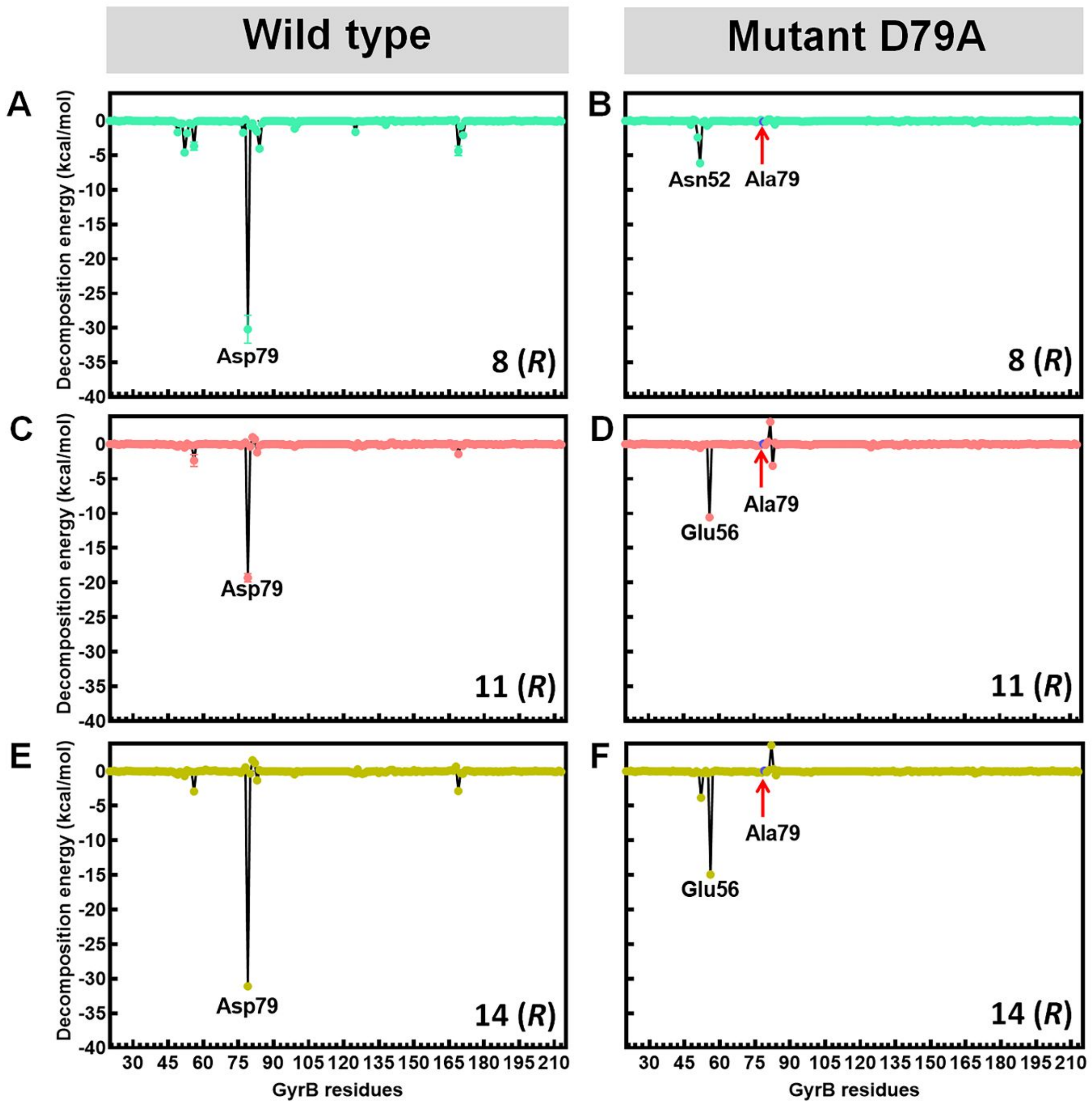

Figure S3. Per-residue analysis of inhibitor:GyrB (ATPase domain) interactions. (A), (C), and (E) Plots of decomposition energies showing contributions of individual residues to binding of compounds **8(*R*)**, **11(*R*)**, and **14(*R*)** to wild-type GyrB. (B), (D), and (F) Plots of decomposition energies showing contributions of individual residues to binding of compounds **8(*R*)**, **11(*R*)**, and **14(*R*)** to mutant GyrB (D79A).

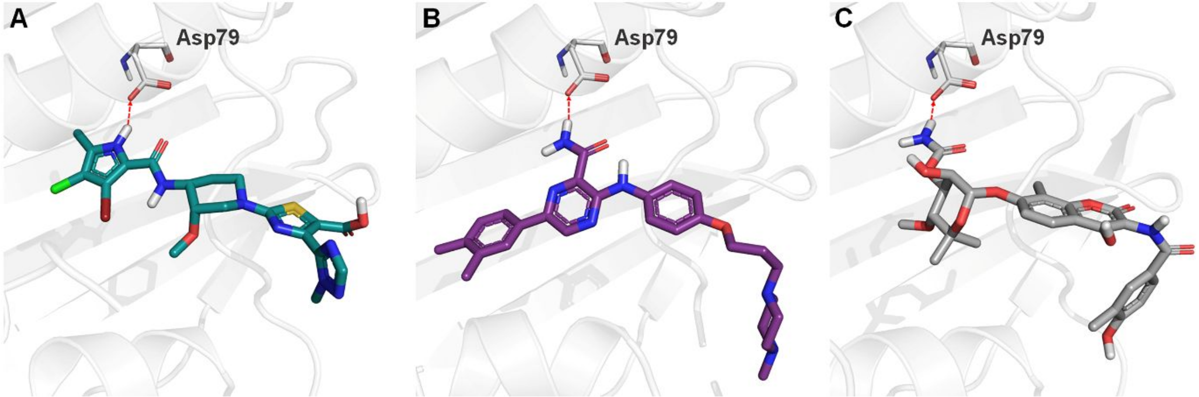

Figure S4. Hydrogen bond interactions (dashed red arrows) of known ATPase inhibitors with Asp79 in the *M. smegmatis* DNA gyrase ATP binding site. (A) pyrrolamide (Deepteal sticks), (B) aminopyrazinamide (Deeppurple sticks), and (C) novobiocin (gray sticks) obtained from PDB codes 4BAE[24], 6Y8O[31] and 4B6C[59], respectively.

Table S1. Hits and leads obtained from three different virtual screening methods

Methods	Hits	Leads active against *M. tuberculosis* H37Ra	Leads active for inhibition of ATPase activity	Leads active against both *M. tuberculosis* and ATPase activity
Ligand-based and structure-based virtual screening (previous method)[32]	9	0	7	0
Percentage of hits to leads		0%	78%	0%
Structure-based virtual screening (previous method)[33]	30	2	2	2
Percentage of hits to leads		7%	7%	7%
Ligand-based virtual screening (current method)	14	10	6	6
Percentage of hits to leads		71%	43%	43%

Table S2. Previously characterized compounds identified from virtual similarity screening. List of fourteen compounds (Specs ID, chemical structure) with *M. tuberculosis* growth inhibitory activity (three active, eleven inactive) as reported on PubChem.

Specs ID	Chemical structure	IC_{50} [a]
AF-399/41048884	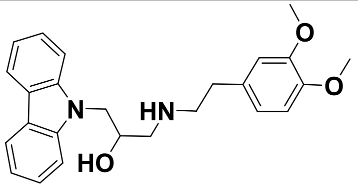	10.61 µM[1] (*M. tuberculosis* H37Rv)
AG-690/11353646	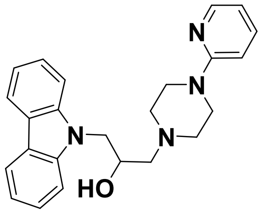	36.12 µM[2] (*M. tuberculosis*)
AP-124/41376194	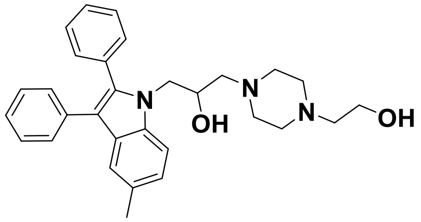	1.84 µg/mL[3] (*M. tuberculosis* H37Rv)
AE-641/05526042	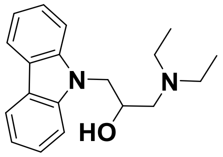	Inactive (*M. tuberculosis* H37Rv)
AE-641/05526043	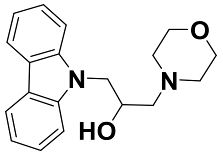	Inactive (*M. tuberculosis* H37Rv)
AG-205/06683048	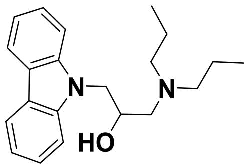	Inactive (*M. tuberculosis* H37Rv)
AG-205/06683053	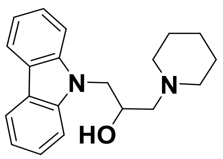	Inactive (*M. tuberculosis*)

AG-205/40649607	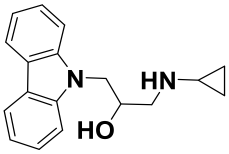	Inactive (*M. tuberculosis* H37Rv)
AG-690/12411004	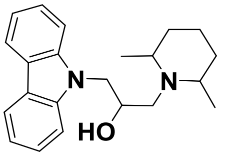	Inactive (*M. tuberculosis*)
AG-690/13153176	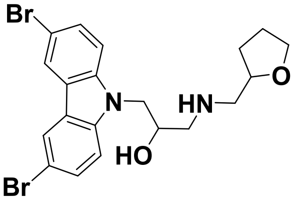	Inactive (*M. tuberculosis* H37Rv)
AG-690/33352020	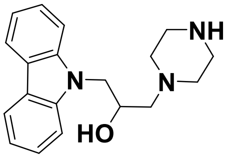	Inactive (*M. tuberculosis* H37Rv)
AP-083/15505010	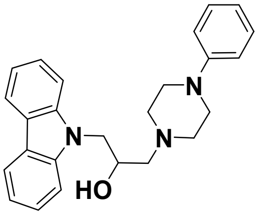	Inactive (*M. tuberculosis* H37Rv)
AP-124/42316482	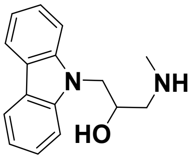	Inactive (*M. tuberculosis* H37Rv)
AS-871/41069852	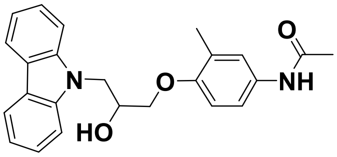	Inactive (*M. tuberculosis* H37Rv)

[a] Biological activity as reported on PubChem

Table S3. Details of fit parameters for IC_{50} determinations shown in Figure 3

Compound	IC_{50} (μM)	Confidence Interval (95 %)	R-squared
**1**[a]	0.73[a]	0.606 – 0.875[a]	0.9724[a]
**2**	1.34	1.120 – 1.690	0.9979
**3**	3.82	2.740 – 6.440	0.9977
**8**	0.26	0.204 – 0.370	0.9940
**11**	0.56	0.462 – 0.707	0.9953
**14**	0.08	0.066 – 0.086	0.9979
**G24**	2.69	2.130 – 3.820	0.9944
**Novobiocin**	1.27	1.060 – 1.540	0.9945

[a]for compound **1**, note that reported values were obtained from fits where the Hill coefficient was fixed at 1. This was varied for all other data sets.

Table S4. Hydrogen bonding of most active compounds (*R-stereomers*) to GyrB Asp79. Percentage occupancy, distance and angle of hydrogen bonds made by compounds **8**, **11**, and **14** were obtained from MD simulations. A donor-acceptor distance of less than 3.5 Å, and an angle between hydrogen bond donor and acceptor of more than 120°, were used as the criteria for hydrogen bond analysis.

Compound	Hydrogen bond		Occupancy (%)	Distance (Å)	Angle (º)
**8**(*R*)	8(*R*)@O**H**	Asp79@**O**D1	100	2.6 ± 0.1	163
	8(*R*)@N**H**(1)	Asp79@**O**D2	100	2.9 ± 0.1	161
	8(*R*)@N**H**(2)	Asp79@**O**D2	97	2.7 ± 0.1	149
**11**(*R*)	11(*R*)@N**H**	Asp79@**O**D2	70	2.9 ± 0.2	150
	11(*R*)@O**H**	Asp79@**O**D1	69	2.8 ± 0.3	159
**14**(*R*)	14(*R*)@O**H**(1)	Asp79@**O**D1	98	2.6 ± 0.1	167
	14(*R*)@N**H**	Asp79@**O**D2	99	2.8 ± 0.1	163
	14(*R*)@O**H**(2)	Asp79@**O**D2	99	2.6 ± 0.1	164

Table S5. Calculated binding free energies (ΔG_{GBSA} and ΔG_{PBSA}) for binding of compounds **8**, **11**, and **14** to the wild type and mutant (D79A) GyrB subunits.

**Compound**	**ΔG_{GBSA} (kcal/mol)**			**ΔG_{PBSA} (kcal/mol)**		
	**Wild type**	**D79A**	**Δ**	**Wild type**	**D79A**	**Δ**
**8**^{R}	−59.1	−38.7	20.4	−48.3	−41.9	6.4
**11**^{R}	−62.3	−47.8	14.5	−56.2	−49.4	6.8
**14**^{R}	−72.3	−48.2	24.1	−62.3	−52.4	9.9

REFERENCES

1. National Center for Biotechnology Information. PubChem Bioassay Record for Bioactivity AID 1626 – SID 24803304, Source: SRMLSC. https://pubchem.ncbi.nlm.nih.gov/bioassay/1626#sid=24803304. Accessed Dec. 15, 2023.

2. National Center for Biotechnology Information. PubChem Bioassay Record for Bioactivity AID 449762 – SID 852599, Source: Southern Research Specialized Biocontainment Screening Center. https://pubchem.ncbi.nlm.nih.gov/bioassay/449762#sid=852599. Accessed Dec. 15, 2023.

3. National Center for Biotechnology Information. PubChem Bioassay Record for Bioactivity AID 1949 – SID 49774400, Source: Southern Research Institute. https://pubchem.ncbi.nlm.nih.gov/bioassay/1949#sid=49774400. Accessed Dec. 15, 2023.
